# Education in Iowa

**Published:** 1854-02

**Authors:** 


Education in Iowa.—There is one thing, which, more than any other, indicates the rapid, favorable and permanent progress of our young State. We refer to the eminently successful establishment of the Iowa Journal of Education, published at Dubuque, having for its object the promotion of Educational interests throughout the State.

We do not hesitate to affirm that no enterprize ever originated within the limits of Iowa is so important to the State, or contains the elements of so durable and universal prosperity. The old Greeks and Romans had liberal views of education. But we pledge our fortunes to new and sometimes rival lines of Rail Roads and steamers, while if efforts are made to build up, or strengthen and enlarge the basis of popular enlightenment, among us, most men content themselves with thinking that perhaps it may be well enough—Happily, however, the Iowa Educational Journal does not need any efforts of ours in its support, but the interests of the community demand the exertions of every one.

No labored homilies are needed, in this age, respecting the importance of education—Every department of art and industry depends more or less upon the broad basis of popular enlightenment. How plain the fact that the professions are all based upon it. Where shall we find a successful ministry, a learned system of law, or an able ank skilful body of medical men, if the community its self be ignorant and degraded? We may justly affirm that popular education should stand among the monument of the day, even before the profession themselves; for what are they without it? We are earnest in our zeal that medical men, may feel an interest in this subject. In a new coountry like, every effort should be made to urge forward so important a cause. That physician does only a part of his duty, who confines his labors to the bedside of suffering—he is not only a physician, but a neighbor, a citizen, an American. He passes daily by that temple of our national liberity, the school house, and whatever concerns the progress of the human race, concerns him.

